# Observations on the Coexistence of Variola and Scarlatina, with Remarks on the Coexistence of Other Eruptive Fevers

**Published:** 1847-09

**Authors:** J. F. Marson

**Affiliations:** Surgeon to the Small-Pox and Vaccination Hospital, London


*Observations on the coexistence of Variola and Scarlatina, with remarks on the coexistence of other eruptive fevers.* By J. F. Marson, Surgeon to the Small-Pox and Vaccination Hospital, London.—During the last eleven years, the author of this paper has seen, at the Small-Pox and Vaccination Hospital, seven persons who had variola and scarlatina simultaneously. These patients were apparently suffering from small-pox only on their admission, but in the course of a few days scarlet fever also developed itself. In each case, all the leading symptoms of scarlatina were well marked, and the eruption was evidently different from the roseola which frequently precedes the eruption of small-pox, and also different from the erythema, (somewhat resembling it,) arising from the miasm of hospitals; in fact, it was the florid red eruption peculiar to scarlatina, which no other eruption exactly resembles. Three of the patients were unprotected, and four of them had been vaccinated. All recovered but one, the particulars of whose case were given in full. Three other patients with variola and scarlatina existing at the same time, have been seen, within the last few years, at the London Fever Hospital. Reference was made to the opinion so strongly expressed by Mr. Hunter, that no two fevers could be coexistent. Several cases were then alluded to, that have been published by different observers, in France and England, of the coexistence of variola and scarlatina, variola and rubeola, variola and pertussis, variola and vaccinia; rubeola and scarlatina, rubeola and vaccinia, rubeola and pertussis; varicella and vaccinia, pertussis and vaccinia. The individuality of erysipelas, as a special eruptive fever, was commented on, this disease being shown to arise, almost invariably in hospitals, from the impure air produced by morbid animal effluvia. The French were acquainted with the fact, fifty years ago, of small-pox and scarlatina existing together occasionally, some cases being referred to by the author, published by M. G. Vieusseux, at that period, but the subject has nearly escaped attention, or at least remark, by writers of this country.—*Dub. Med. Press.*

---

Sir Astley Cooper was kind enough to make us acquainted with his researches on the structure and functions of the thymus gland, with which he was then occupied; and I am the more pleased to recall this circumstance, because it enables me to record a reply of Sir Astley's, which proves delightfully the perfect truth and honesty with which he conducts his researches and experiments. While he was pointing out to us, on a most delicate preparation, the two membranes which he has found in what he calls the reservoirs of the thymus, I said to him, "You said, and it is." "No," he replied, "It is, and I said." The scientific character of the great English surgeon breathes in this response.—*Prov. Med. and Surg. Journ. from Six mois de Sejour en Angleterre par S. Pironde, D. M.—British and Foreign Medical Review*, vol. 8, p. 534.

---

When Dr. Dimsdale inoculated Catherine the Second for the small-pox, that Princess (who, whatever might have been the vices of her moral character, possessed a very large and magnanimous mind,) took precautions for securing her personal safety in case of her death. Finding herself much indisposed on a particular day, she sent for Dimsdale, whom she had already remunerated in a manner becoming so great a sovereign. "I experience," said she, "certain sensations which render me apprehensive for my life. My subjects would, I fear, hold you accountable for any accident that might befall me. I have therefore stationed a yacht in the Gulf of Finland, on board of which you will embark, as soon as I am no more, and whose commander, in consequence of my orders, will convey you out of all danger." This anecdote, so honourable to the Empress, I heard from one of Dimsdale's sons, above forty years ago.—*Ibid, from Sir N. W. Wraxall's Posthumous Memoirs of his own Times*, vol. 3, p. 199.

